# *Bacillus safensis* APC 4099 has broad-spectrum antimicrobial activity against both bacteria and fungi and produces several antimicrobial peptides, including the novel circular bacteriocin safencin E

**DOI:** 10.1128/aem.01942-24

**Published:** 2024-12-31

**Authors:** E. Kamilari, P. M. O'Connor, F. Miceli de Farias, C. N. Johnson, C. Buttimer, A. Deliephan, D. Hill, O. Fursenko, J. Wiese, C. Stanton, C. Hill, R. P. Ross

**Affiliations:** 1 School of Microbiology, University College Cork 219553 , Cork, County Cork, Ireland; 2 APC Microbiome Ireland, Cork, Ireland; 3 Teagasc, Moorepark Food Research Centre, Fermoy, Co., Cork, Ireland; 4 Department of Biochemistry & Microbiology, Center for Health Sciences, Oklahoma State University 33264 , Tulsa, Oklahoma, USA; 5 Kraft Heinz Corporate Headquarters, Chicago, Illinois, USA; The Pennsylvania State University, University Park, Pennsylvania, USA

**Keywords:** *Bacillus safensis*, antimicrobial peptides, circular bacteriocin, safencin E, pumilarin, plantazolicin, pumilacidin, bacilysin, bacillibactin, food biopreservation

## Abstract

*Bacillus safensis* APC 4099, isolated from bees’ gut, has been identified as a promising candidate for food biopreservation. Antimicrobial activity screening revealed a broad-spectrum inhibition potential, ranging from gram-positive pathogenic bacteria to fungi responsible for food spoilage. Genomic analysis identified biosynthetic gene clusters coding for several antimicrobial peptides and secondary metabolites. Specifically, a novel, anionic, 6 kDa circular bacteriocin, named safencin E, was detected, showing 52.5% similarity to butyrivibriocin AR10. Additionally, gene clusters coding for the biosynthesis of bacteriocins such as pumilarin and plantazolicin and biosynthetic pathways for secondary metabolites, including pumilacidin A, bacilysin, and bacillibactin, were identified. Matrix-assisted laser desorption ionization-time of flight mass spectrometry analysis detected molecular masses correlating to safencin E, plantazolicin, pumilarin, and pumilacidin A from the cell-free supernatant, cell extracts, or both. Overall, the broad-spectrum antimicrobial activity of *B. safensis* APC 4099 indicates that this strain is a promising candidate for the biological control of food ecosystems and thus has the potential to enhance food safety.

**IMPORTANCE:**

The present article highlights the importance of the strain *Bacillus safensis* APC 4099 as a potential biocontrol agent. The strain possesses biosynthetic gene clusters coding for various antimicrobial peptides and secondary metabolites, including a novel circular bacteriocin, safencin E, and the bacteriocins pumilarin and plantazolicin. This diversity in the production of antimicrobial peptides renders the producer with broad-spectrum antimicrobial activity, ranging from gram-positive pathogenic and spoilage bacteria to spoilage molds. Considering that 1.3 billion tons of food appropriate for human consumption is lost or wasted annually, identifying strains or novel antimicrobial peptides capable of biopreservation is highly relevant. This strain and its bioactive compounds offer a solution to this global problem as biocontrol agents for food ecosystems against spoilage and pathogenic microbes.

## INTRODUCTION

Food biopreservation is among the current sustainable solutions for extending shelf life and improving the safety of food products (1, 2). This promising approach involves the administration of safe, natural, or controlled microbial cultures or their antimicrobial compounds. A fermentation process guided by the presence of beneficial microbes, or their bioactive compounds, prevents the growth of pathogenic and spoilage microbes and suppresses the release of undesired metabolites that spoil the quality of the food product (3). Bacteriocins are considered notable candidates for shaping the food microbiome (4). These ribosomally-synthesized peptides can prevent the colonization of specific microbes, typically those closely related to the bacteriocin producer (5). Currently, the bacteriocins nisin (Nisaplin; Danisco, Dupont), NisinA/Z and NisinA/Z P (Handary), Chrisin (Chr. Hansen), Pediocin PA-1 (ALTA 2351/2341; Kerry Group), Fargo 23 (Quest International), and carnocyclin A (Micocin, Griffith Foods), are used commercially for food safety applications (6–8).

*Bacillus* species are gram-positive, rod-shaped bacteria that form endospores and can be detected in several environments, including soil, food, aquatic environments, air, and the gastrointestinal tracts of arthropods and mammals (9). Many strains of *Bacillus* are generally recognized as safe by the Food and Drug Administration. Also, several *Bacillus* species can produce various antimicrobial substances, such as bacteriocins and lipopeptide antibiotics. Based on post-translational modifications, bacteriocins are divided into two classes (10–12). Class I bacteriocins, or lantibiotics, are small peptides (≤5 kDa) that contain serine, threonine, and cysteine amino acid residues, on which post-translational modifications are performed. Class II bacteriocins are small, linear peptides (≤10 kDa) without post-translational modification. Circular bacteriocins have been recently reclassified to Class I bacteriocins, because of the post-translational circularization created by a covalent bond between the N- and C-terminal amino acids (8). Circular bacteriocins are subdivided into two groups based on their biochemical characteristics. Group I peptides possess mainly cationic residues and high isoelectric points (pI > 9), while Group II peptides have increased hydrophobic, acidic residues and a lower pI than Group I (pI < 7). Their mode of action involves a direct interaction of the circular bacteriocin with the target bacterial cell membrane, which leads to cell permeability, causing a leakage of ions. As a result, the membrane potential is disrupted, leading to cell death (13). Apart from bacteriocins, *Bacillus* species produce other secondary metabolites, such as polyketides, terpenes, siderophores, and other ribosomally and non-ribosomally synthesized peptides. Furthermore, bacilli are well known for biosurfactant production, including cyclic lipopeptides, such as lichenysins, bacillomycin, fengycins, and surfactins (14).

Bacteriocins produced by *Bacillus* species have the potential to be used in several biotechnological applications, including medical and veterinary applications and food biopreservation (15–18). For instance, the bacteriocins CAMT2, bacthuricin F103, and bacicyclicin XIN-1 produced by *B. amyloliquefaciens* ZJHD3, *B. thuringiensis*, and *Bacillus* sp. Xin1, respectively, demonstrated anti-*Listeria monocytogenes* activity in pork meat (19), minced beef (20), and skim milk (21), respectively. Additionally, the bacteriocin BpSl14 produced by *B. safensis* stimulated apoptosis in A549 human lung carcinoma cells, preventing their proliferation (22). Moreover, bacitracin, which is an antibiotic, produced by *Bacillus licheniformis* has been approved for clinical use against skin and eye infections (12). Furthermore, *Bacillus* lipopeptides have been applied in several food products to enhance their antimicrobial, surfactant, and emulsifying characteristics (14). Specifically, lipopeptides from *Bacillus subtilis* reduced contamination of the fungus *Aspergillus carbonarius* and the ochratoxin A it produces, which is considered a carcinogenic mycotoxin at concentrations above 2.0 µg/L and is responsible for enhancing acid and ester production during winemaking (23).

In this study, we combined antimicrobial activity screening, genomic sequencing and matrix-assisted laser desorption ionization-time of flight mass spectrometry (MALDI-TOF MS) analysis to identify and characterize the broad-spectrum antimicrobial activity of *B. safensis* APC 4099, a strain isolated from bees’ gut. We show that *B. safensis* APC 4099 produces a range of antimicrobial compounds including bacteriocins and secondary metabolites, the former of which includes a novel circular bacteriocin, named safencin E.

## MATERIALS AND METHODS

### Bacterial strain and culture conditions

*B. safensis* APC 4099 (KH-2006), was isolated from the honeybees’ (*Apis mellifera*) gut obtained from Kerry in Ireland. The strain was selected after screening 88 isolated strains for antimicrobial activity against specific microbial indicators, as shown in (Table 1). The strain was cultured in tryptic soy broth (TSB, Merck, Germany) and incubated aerobically at 30°C, under shaking at 200 rpm. Genomic DNA was extracted using the DNeasy PowerFood Microbial Kit (MoBio Laboratories Inc., Carlsbad, CA, USA), according to the manufacturer’s instructions. Identification of strain APC 4099 as *B. safensis* was performed using Next Generation Sequencing (NGS). Specifically, the genome of *B. safensis* APC 4099 was sequenced by MicrobesNG (Birmingham, UK), using the MiSeq Illumina and Oxford Nanopore sequencing platform. The closest available reference genome was identified using Kraken (24) and the reads were mapped to this using Burrows-Wheeler Aligner (BWA) (25). Genome average nucleotide identity (ANI) calculations were performed using pyani (https://github.com/widdowquinn/pyani).

**TABLE 1:** Antimicrobial activity of *B. safensis* APC 4099 against indicator strains using spot assay (using the strain) and well diffusion assay (using the CFS)^*a*^

Microorganism	Spot assay	Well diffusion assay	Growth conditions
	Zone of inhibition (mm)	Zone of inhibition (mm)	
*Clostridium perfringens* EM 124	23 ± 1	16	37°C, anaerobic
*Clostridium tyrobutyricum* APC 044	20 ± 1	15	37°C, anaerobic
*Bacillus cereus* KH-1453	19 ± 1	14	30°C, aerobic
*Listeria innocua UCC*	14 ± 1	12	30°C, aerobic
*Listeria monocytogenes EDGe*	13 ± 1	11	30°C, aerobic
*Staphylococcus aureus UCC*	20 ± 1	15	30°C, aerobic
*Staphylococcus epidermidis* KM 1928	22 ± 1	12	30°C, aerobic
*Staphylococcus caprae* DSM 20608	12 ± 1	10 ± 1	30°C, aerobic
*Staphylococcus aureus* A8M	14 ± 1	9 ± 2	30°C, aerobic
*Staphylococcus aureus* B2M	0	0	30°C, aerobic
*Staphylococcus aureus* DPC 5645	12 ± 1	11	30°C, aerobic
*Staphylococcus aureus* CSM	11 ± 1	10	30°C, aerobic
*Staphylococcus pseudintermedius* DK279	0	0	30°C, aerobic
*Staphylococcus epidermidis* DSM 9035	0	0	30°C, aerobic
*Lactobacillus bulgaricus* LMG 6901	16 ± 1	12	37°C, anaerobic
*Leuconostoc mesenteroides* APC 4234 (KH-024)	38 ± 1	8 ± 1	30°C, aerobic
*Lactobacillus helveticus* DPC 5358	0	0	30°C, aerobic
*Lactococcus lactis* ATCC 11454	0	0	30°C, aerobic
*Lactococcus lactis* HP	0	0	30°C, aerobic
*Bavariicoccus seileri* DSM 15936	0	0	30°C, aerobic
*Enterococcus faecalis* ATCC 29200	0	0	30°C, aerobic
*Enterococcus faecalis* KH-157	0	0	30°C, aerobic
*Enterococcus faecalis* DSM26544	0	0	30°C, aerobic
*Enterococcus faecalis* VRE	0	8	30°C, aerobic
*Enterococcus faecalis* NCD 0942	0	0	30°C, aerobic
*Enterococcus faecalis* DPC 3675	0	0	30°C, aerobic
*Micrococcus luteus* APC 4061	0	0	30°C, aerobic
*Micrococcus lactis* DSM 1730	0	0	30°C, aerobic
*Escherichia coli* UCC	0	0	30°C, aerobic
*Klebsiella pneumoniae* UCC	0	0	30°C, aerobic
*Pseudomonas aeruginosa* UCC	0	0	30°C, aerobic
*Zygosaccharomyces bailii* strain Sa-1403	0	0	30°C, aerobic
*Yarrowia lipolytica* strain 78–003	0	0	30°C, aerobic
*Saccharomyces cerevisiae* type strain Sa-07140	0	0	30°C, aerobic
*Aspergillus niger* UCC	28 ± 1	10 ± 3	30°C, aerobic
*Byssochlamys nivea* UCC	18 ± 1	10 ± 2	30°C, aerobic
*Geotrichum* sp. UCC	12 ± 1	8 ± 1	30°C, aerobic
*Paecilomyces variotii* UCC	20 ± 1	11 ± 1	30°C, aerobic
*Cladosporium* sp. UCC	18 ± 1	10 ± 1	30°C, aerobic
*Phoma* sp. UCC	0	0	30°C, aerobic
*Penicilium notatum* UCC	0	0	30°C, aerobic

^*a*^ All indicators were sourced from APC Microbiome, University College Cork. Data are indicated as mean ± standard deviation (*n* = 3).

### Anti-bacterial and anti-fungal activity assay

*B. safensis* APC 4099 was tested for antagonistic activity against lactic acid bacteria (LAB), spoilage and pathogenic bacteria, yeast, and mold indicators, using the spot-on lawn technique (26). Specifically, 1 µL of 24 h grown culture of *B. safensis* APC 4099 was spotted on tryptic soy agar (TSA, Merck, Germany) and incubated at 30°C for 24 h. Following growth, the plates were overlaid with 10 mL of 0.75% w/v “sloppy” agar of temperature 50˚C, containing 10^5^ to 10^6^ colony forming units (CFU)/mL bacterial culture, and 10^5^ to 10^6^ fungal spores (Table 1). After incubation at 30°C or 37°C for 24 h to test bacteria and 24-72 h to test fungi, zones of inhibition surrounding the *B. safensis* APC 4099 colony spot were measured. All indicator species were sourced from the culture collection maintained by APC Microbiome Ireland at University College Cork. The overlaid “sloppy” agar was comprised of: (i) BD Difco *Lactobacilli MRS*, Thermo Fisher Scientific, Denmark (MRS) for LAB; (ii) *Reinforced Clostridial Medium, Oxoid, UK for Clostridia sp.;* (iii*) TSA*, Merck, Germany for additional gram-positive and gram-negative bacteria; and (iv) *Sabouraud dextrose agar* (SDA) for molds. Antibacterial and antifungal activity were evaluated according to the diameter of the inhibition zone.

### Optimization of the inhibitory activity

Initially, the antimicrobial activity protocol was optimized by performing growth kinetics of the strain using different media. These included the supplementation of TSB medium with different carbon sources at a concentration of 3% (glucose [Merck, Germany], sucrose [Merck, Germany], lactose [Merck, Germany], fructose [Merck, Germany] and glycerol [Merck, Germany]) and 1.5% different nitrogen sources (tryptone [Merck, Germany] and yeast extract (Fisher Scientific, UK)), and addition or not of extra 0.1% casamino acids (Merck, Germany), 0.2% magnesium sulfate heptahydrate [Fisher Scientific, UK], and 1% sodium chloride (Merck, Germany). *B. safensis* APC 4099 was inoculated (1% inoculum) in 500 mL of each medium and incubated aerobically at 30°C, under shaking at 200 rpm. Every 4 h for 48 h, 10 mL from the growing culture was collected and centrifuged at 5,000×*g* at 4°C for 20 min. The supernatant was passed through a 0.2 μm sterile filter (Sarstedt AG & Co, Numbrecht, Germany) and the cell-free supernatant (CFS) was collected. The cell pellet was treated with 1 mL 70% (vol/vol) propan-2-ol-containing 0.1% (vol/vol) trifluoroacetic acid (TFA). After incubation at 30°C for 3 h under shaking conditions and centrifugation at 4,000×*g* at 4°C for 10 min, the CFS was collected as described above. In order to evaluate the inhibitory activity of the CFS, the well diffusion assay (WDA) was used, whereby 50 mL 0.75% wt/vol “sloppy” agar was inoculated with 10^5^ to 10^6^ CFU/mL of bacteria (as determined spectrophotometrically at OD_600_), and 10^5^ to 10^6^ spores/mL of fungi (spores of the germinating fungus were counted via optical microscopy) and poured into a square Petri dish. Using a glass Pasteur pipette sterilized with 70% ethanol (VWR International Limited, cat no. 612-3813), 6 mm diameter wells were created in the agar plate. In each well, 50 μL of CFS was incorporated, and the plate was incubated at the appropriate temperature for 24–72 h depending on the indicator species tested. Antimicrobial activity, as observed by a clear zone due to lack of microbial growth around the well, was measured in millimeters corresponding to the zone diameter. The medium and the time point with the highest inhibition zone for each indicator were recorded.

### Growth of microbial indicators in different concentrations of CFS

Following selection of the medium with the greatest antimicrobial activity, *B. safensis* APC 4099 was inoculated (1% inoculum) in 1L of TSB supplemented with 3% lactose, 1.5% tryptone, 0.1% casamino acids, 0.2% magnesium sulphate heptahydrate, and 1% sodium chloride and incubated aerobically at 30°C for 48 h, under shaking at 200 rpm. The CFS was collected as described above and evaluated against the microbial indicators (Table 1) as a dose-dependent response. Using 10^5^ to 10^6^ CFU/mL inoculum, the bacterial and fungal indicators were grown in the presence of 0% (control), 5%, 10% 25%, and 50% CFS. Experiments were performed in triplicate. The inhibition curves of indicator strains were determined using a microplate reader at a wavelength of 590 nm, OD_590nm_ (Multiskan Fc model; Thermo Scientific, Loughborough, UK), and were displayed using the SkanIt Software for Microplate Readers RE, ver 7.0.0.50. Statistically significant differences based on one-way analysis of variance were evaluated using the IBM SPSS software based on the least significant difference (LSD) at the significance level of 0.05.

### Bioinformatics analysis

Raw reads were assembled using SPAdes v3.14.0 using the MEGAnnotator pipeline (27). Genome annotation was performed using Prokka (Galaxy version 1.14.6) (28), and NCBI Prokaryotic Genome Annotation Pipeline (PGAP) (29). The identification of open reading frames (ORFs) was performed using Prodigal v2.6, and their automatic annotation using RAPSearch2 against the NCBI RefSeq database and HMMER against the PFAM database. Prediction of variance relative to reference was achieved with VarScan (30). Prediction of ribosomal RNA genes and transfer RNA genes was achieved with RNAmmer v1.2. and tRNAscan-SE v1.21, respectively. The BAGEL4 (31) and antiSMASH v.7.0 (32) databases were used to predict bacteriocin/antimicrobial/secondary metabolites gene clusters. Additional manual annotation was performed using ARTEMIS and Artemis Comparison Tool. Non-ribosomally produced peptide (NRP) gene clusters were visualised using CAGECAT (https://cagecat.bioinformatics.nl/). Sequence alignments of the bacteriocin were performed using Clustal Omega (33) and T-Coffee (34) software and visualized using WebLogo (version 3.0) (35). Phylogenetic trees were generated with PhyML (36) available at the Montpellier bioinformatics platform (http://www.atgc-montpellier.fr/phyml/), based on the maximum-likelihood algorithm. An unrooted tree was designed based on the Newick format using the TreeDyn tool (Phylogeny fr: robust phylogenetic analysis for the non-specialist). Secondary structure predictions of the identified bacteriocins were performed using the I-Tasser protein structure and function predictions server (37, 38) and visualized using Chimera X (39, 40). The theoretical molecular mass and isoelectric point (pI) of amino acid sequences were predicted using an online platform (https://web.expasy.org/compute_pi/). The cellular localization for the encoded protein was predicted using TMHMM (https://dtu.biolib.com/DeepTMHMM/). The Galaxy platform ABRicate v. 1.0.1 was used to identify the presence of antibiotic resistance, antimicrobial and virulence genes (41). The genome sequence was deposited in GenBank under the accession number JAWDAB00000000 (https://www.ncbi.nlm.nih.gov/nuccore/JAWDAB000000000) in the bioproject PRJNA963206.

### Colony MALDI-TOF mass spectrometry

Initially, a *B. safensis* APC 4099 cell extract was assessed for the presence of peptide molecular masses correlating with the known molecular mass of antimicrobials expressed by the strain. A loop full of *B. safensis* APC 4099 colonies grown on BHI agar was mixed with 50 µL of 75% (vol/vol) propan-2-ol containing 0.1% TFA (IPA). The mixture was vortexed three times. The suspended cells were centrifuged, and the IPA extract was retained for MALDI-TOF MS (matrix-assisted laser desorption/ionisation coupled to time-of-flight mass spectrometry) analysis by an iD^plus^ Performance MALDI-TOF mass spectrometer (Shimadzu, Duisburg, Germany). Specifically, a 0.5 µL aliquot of matrix solution (α-cyano 4-hydroxy cinnamic acid, 10 mg/mL in acetonitrile containing 0.1% [vol/vol] trifluoroacetic acid) was applied to the target plate, allowed to dry for 20 s before excess solvent was discarded. The sample was placed on the pre-coated sample spot and 0.5 µL of matrix solution was added. Once dry, the solution was analyzed in positive-ion linear mode.

### Purification and identification of the antimicrobials produced by *B. safensis* APC 4099

*B. safensis* APC 4099 was inoculated into 200 mL of TSB containing 0.5% lactose and cultivated at 37°C for 72 h, at a stirring speed of 200 rpm. Following centrifugation at 8000 rpm (5,734 × *g*) at 10°C for 20 min, the CFS was separated from the cells. The CFS was passed through a 5g (20 mL) Strata C18-E SPE column (Phenomenex, Cheshire, UK) pre-equilibrated with methanol and water. After a washing step with 30 mL of 40% ethanol, the antimicrobials were eluted using 20 mL of 75% (vol/vol) IPA.

The cell pellet was mixed with 50 mL of 75% IPA, stirred at room temperature for 3-4 h to extract the antimicrobials from the cell membrane and re-centrifuged. The IPA was removed from the cell extract via rotatory evaporation and the sample passed through a 5 g (20 mL) Strata C18-E SPE column as described for the CFS above.

Aliquots of semi-purified sample from the CFS and cell extract were applied to a Jupiter C5 semi-preparative (10 × 250 mm^2^, 5 µm, 300 Å) reversed-phase HPLC column (Phenomenex, Cheshire, UK) running a three-way gradient of 5–55% buffer B and 0–5% buffer C over 25 min followed by 55–19% buffer B and 5–65% buffer C over 60 min, 19–5% buffer B and 65–95% buffer C over 5 min where mobile phase A was 0.1% TFA, mobile phase B was 100% acetonitrile and 0.1% TFA, and mobile phase C was 100% propan-2-ol and 0.1% TFA. Eluent was monitored at 214 nm and fractions were collected at 1-min intervals. The active fractions were assayed for the antimicrobial masses of interest using MALDI-TOF MS.

## RESULTS

### Antibacterial and antifungal activity of *B. safensis* APC 4099 culture and cell-free supernatant

To evaluate the antibacterial and antifungal activity of *B. safensis* APC 4099, spot-assays were initially performed against 41 indicators, including LAB, spoilage and pathogenic bacteria, yeasts, and molds (Table 1). The greatest antibacterial activity using the spot assay was demonstrated against *Leuconostoc mesenteroides* APC 4234 (38 ± 1 mm), followed by clostridia species, including *C. perfringens* EM 124 (23 ± 1 mm) and *C. tyrobutyricum* APC 044 (20 ± 1 mm), and staphylococci species, including *Staphylococcus aureus* UCC (20 ± 1 mm) and *Staphylococcus epidermidis* KM 1928 (22 ± 1 mm). Anti-fungal activity was observed against *Paecilomyces variotii* UCC (20 ± 1 mm)*, Cladosporium* sp. UCC (18 ± 1 mm), *Geotrichum sp*. UCC (12 ± 1 mm), *Aspergillus niger* UCC (28 ± 1 mm), and *Byssochlamys nivea* UCC (18 ± 1 mm) (Fig. S1).

**Fig 1:**
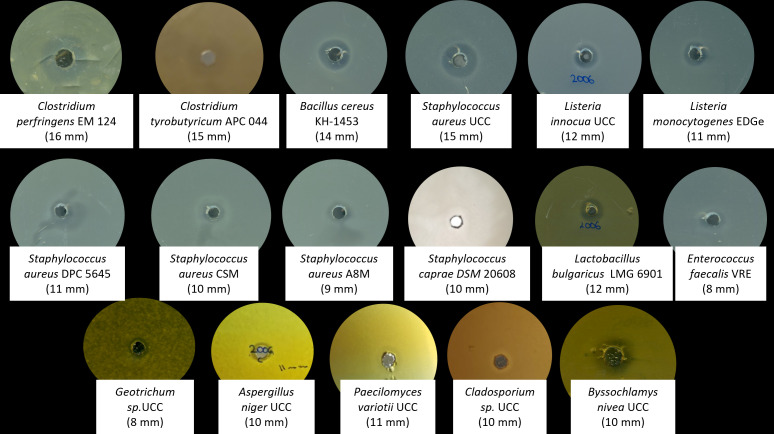
Well diffusion assay of *B. safensis* APC 4099 CFS against a selection of bacterial and fungal indicator strains.

The CFS of *B. safensis* APC 4099 was assessed for antimicrobial production against the indicators listed above using the well-diffusion assay (Fig. 1). The CFS demonstrated inhibitory activity against 11 spoilage and pathogenic bacteria, as well as five molds. A strong inhibitory activity was observed against clostridia (15–16 mm) and staphylococci species, such as *S. aureus* UCC (15 mm) and *S. epidermidis* KM-1928 (12 mm). In addition, the CFS of the strain was active against *L. innocua* (12 mm), *L. monocytogenes* (11 mm)*, L. mesenteroides* APC 4234 (8 mm), *L. bulgaricus* LMG 6901 (12 mm), and *B. cereus* KH-1453 (14 mm). Furthermore, as shown in Table 1, activity against *Geotrichum* sp. (8 mm), *A. niger* (10 mm), *P. variotii* (11 mm), *Cladosporium* sp. (10 mm), and *Byssochlamys nivea* UCC (10 mm), was detected.

**Fig 2:**
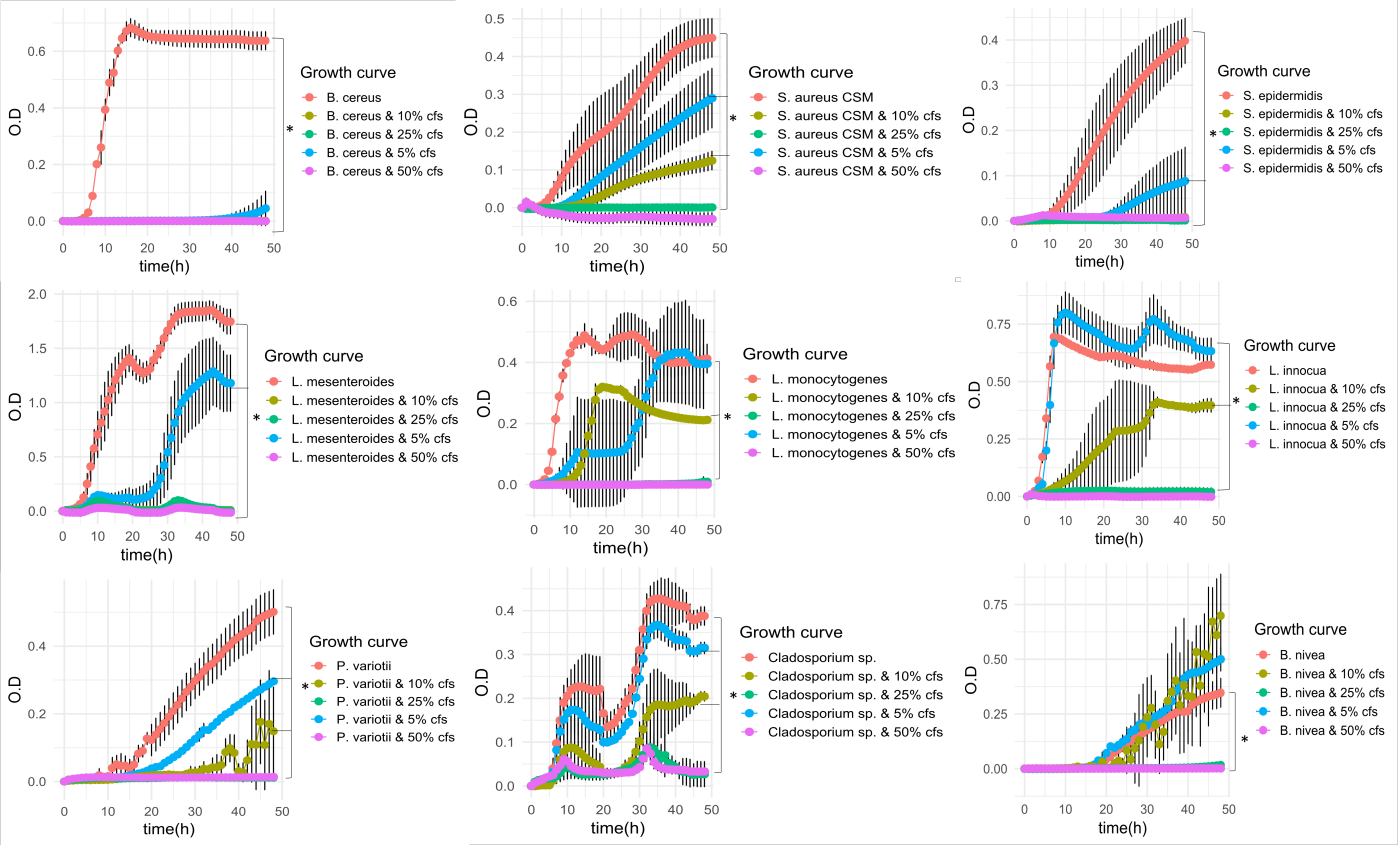
Effect of the active CFS (5%, 10%, 25%, and 50%) and no CFS (control) on the growth of bacterial and fungal indicators, according to the recorded OD_590 nm_ value over time (48 h).

To obtain additional insight into the antimicrobial effects of *B. safensis* APC 4099, the antimicrobial activity protocol was optimized by performing growth kinetics of the strain, by taking samples every 4 h for 48 h, using TSB medium supplemented with 3% different carbon source (glucose, sucrose, lactose, fructose, and glycerol) and 1.5% different nitrogen sources (tryptone and yeast extract), and addition or not of extra 0.1% casamino acids, 0.2% magnesium sulfate heptahydrate, and 1% sodium chloride. The medium and the time point with the highest inhibition zone for each indicator were recorded. Following selection of the medium and time point with the greatest antimicrobial activity, *B. safensis* APC 4099 was inoculated (1% inoculum) in 1 L of TSB supplemented with 3% lactose, 1.5% tryptone, 0.1% casamino acids, 0.2% magnesium sulfate heptahydrate, and 1% sodium chloride and incubated aerobically at 30°C for 48 h, under shaking at 200 rpm. The CFS was collected and used to develop the inhibition curves for each indicator. As observed in Fig. 2, four concentrations of active CFS (5%, 10%, 25%, and 50%) were tested for their ability to prevent the growth of indicator species compared to the control with no CFS added. According to the OD_590nm_ value, 25% and 50% active CFS completely prevented or significantly reduced (*P* ≤ 0.05 based on the LSD; Table S1) the growth of all indicator species tested compared to the control. The CFS was highly active against *B. cereus*, as each of the four concentrations tested prevented the growth of *B. cereus*. Additionally, in the presence of 10% CFS, significant reductions (*P* ≤ 0.05) in the OD_590 nm_ after 24 and 48 h of cultivation were observed for staphylococci spp., including *S. aureus* UCC and *S. epidermidis*, *Listeria* spp., including *L. monocytogenes*, and *L. innocua*, and *L. mesenteroides*. Furthermore, significant reductions in the growth of fungal cultures of *P. variotii, Geotrichum* sp., and *Cladosporium* sp. were observed after 48 h at a concentration of 10% CFS (*P* ≤ 0.05).

### Genomic analysis of *B. safensis* APC 4099 reveals a plethora of bacteriocins and secondary metabolites including a novel class II circular bacteriocin

Whole genome sequence analysis used a combination of short reads (2 × 250 bp Illumina reads) and long reads (Oxford Nanopore) to identify the genes and gene clusters contributing to the broad-spectrum antimicrobial activity of *B. safensis* APC 4099. The assembly resulted in a complete genome with 3,829,124 base pairs, 3,681 predicted ORFs and 46.67% GC content (sequence coverage: short reads 82 and long reads 81). Comparison of the whole genome of *B. safensis* APC 4099 with 11 other complete circular genomes of *B. safensis*, including the type strain (*B. safensis* PgKB20) revealed that the strain exhibits ANI ≥97% (Table S2). The safety of the strain was evaluated using the Galaxy platform ABRicate v.1.0.1, which indicated the absence of virulence factors in the genome (42). However, the gene cat86_1, which encodes a chloramphenicol acetyltransferase that inactivates the drug chloramphenicol (43) was detected. Prokka (Galaxy version 1.14.6) was also used to confirm the absence of antimicrobial and virulence genes (41). Focusing on the major virulence factors that can exist in *Bacillus*, no virulence genes were detected. However, the presence of the gene bslA, an adhesin protein that enables biofilm formation on surface layers, was found (44).

**Fig 3:**
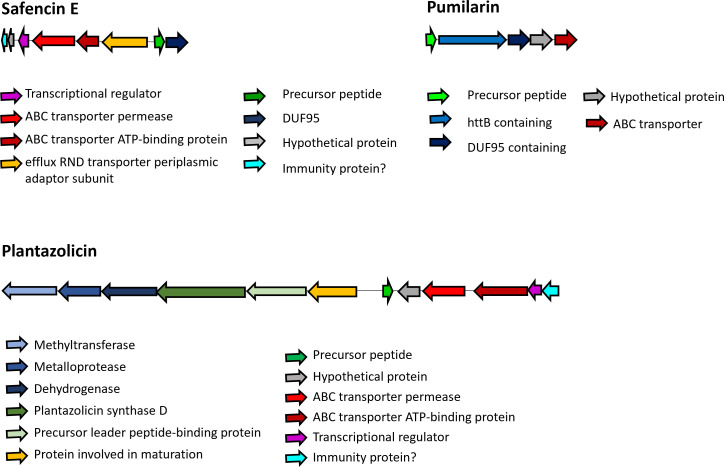
Safencin E, pumilarin, and plantazolicin gene clusters. Genes are color-coded according to the putative role of each protein.

Genome sequence analysis using BAGEL4 and antiSMASH v7.0 revealed that approximately 7.16% of the *B. safensis* APC 4099 genome encodes for several antimicrobial or bioactive compounds (Table 2; Fig. S2). Of particular interest is a bacteriocin-associated gene cluster coding for a novel, 6 kDa circular bacteriocin, hereafter named safencin E, with 52.5% and 47.27% similarity to butyrivibriocin AR10 from *Butyrivibrio fibrisolvens* AR10 and gassericin A from *Lactobacillus gasseri* LA39, respectively (13). The biosynthetic gene cluster of safencin E was predicted to be composed of the structural gene of safencin E precursor (279 bp), a transcriptional regulator, ABC transporters, a DUF95 protein, and a small, unknown function, transmembrane protein, possibly involved in immunity (Fig. 3). In addition to safencin E, a gene cluster predicted to encode for plantazolicin (Fig. 3) was also identified. This gene cluster contains the biosynthetic and immunity genes that were described previously by Scholz and co-workers for plantazolicin produced by *B. amyloliquefaciens* FZB42 (45). These include a DUF3800 domain-containing protein with possible immunity function, a transcriptional regulator, ABC transporters, the precursor peptide, a protein involved in maturation, a thiazole/oxazole-modified microcin (TOMM) precursor leader peptide-binding protein, the plantazolicin synthase D, and the enzymes dehydrogenase, metalloprotease, and methyltransferase. *B. safensis* APC 4099 also contains a gene cluster coding for pumilarin (Fig. 3), initially described in *Bacillus pumilus* B4107 (46), and a cluster involved in a BhlA holin-like peptide expression. The BhlA holin family proteins have been reportedly involved in the non-lytic secretion of folded toxins, endolysins, and bacteriocins from *Clostridium* and *Bacillus* species (47). However, the predicted gene for the BhlA holin-like peptide in *B. safensis* APC 4099 revealed only 37.5% similarity to the one described by Garnier and colleagues (48).

**TABLE 2:** Detected bacteriocins and secondary metabolites in *B. safensis* APC 4099 genome using BAGEL4 and antiSMASH^*a*^

Gene cluster	Genome location		Group	Bacteriocin/secondary metabolite	Similarity percentage	Calculated molecular mass	Percentage of the cluster over the entire genome	Reference
	Start	Finish						
1	509,263	511,077	Terpene	Phytoene/squalene	100%	-	0. 76%	(49)
2	1,122,908	1,204,105	NRPS	Pumilacidin	89%	1,044, 1,058, 1,072, 1,086 Da	2. 19%	(50)
3	1,546,670	1,598,398	NRPS	Bacillibactin	80%	851 Da	1. 39%	(51)
4	1,818,995	1,860,416	Other	Bacilysin	85%	270 Da	1. 11%	(52–54)
5	2,737,465	2,768,136	RiPP	Bottromycin	6%	-	0. 83%	(55)
6	2,903,582	2,903,908	Bacteriocin class IId	Pumilarin	100%	7,091 Da	0. 01%	(46)
7	3,238,004	3,239,098	T3PKS	Unknown	-	-	0. 03%	-
8	3,307,090	3,308,964	Terpene	Squalene-hopene cyclase	-	-	0. 05%	-
9	3,394,232	3,402,641	NRP	Fengycin	53%	1463.8,–1505.8 Da	0. 22%	(56, 57)
10	3,687,955	3,688,935	LAP	Plantazolicin	91%	1336/1354 Da	0. 02%	(45, 58–60)
11	3,701,743	3,702,024	Bacteriocin class IIc	Butyrivibriocin AR10	52. 50%	5998 Da	0. 01%	(61)
12	2,446,292	2,466,496	Holin-like peptides	BhlA/UviB family holin-like peptide	100%	8279 Da	0. 54%	(62, 63)

^*a*^ “-” means Molecular mass unavailable.

**Fig 4:**
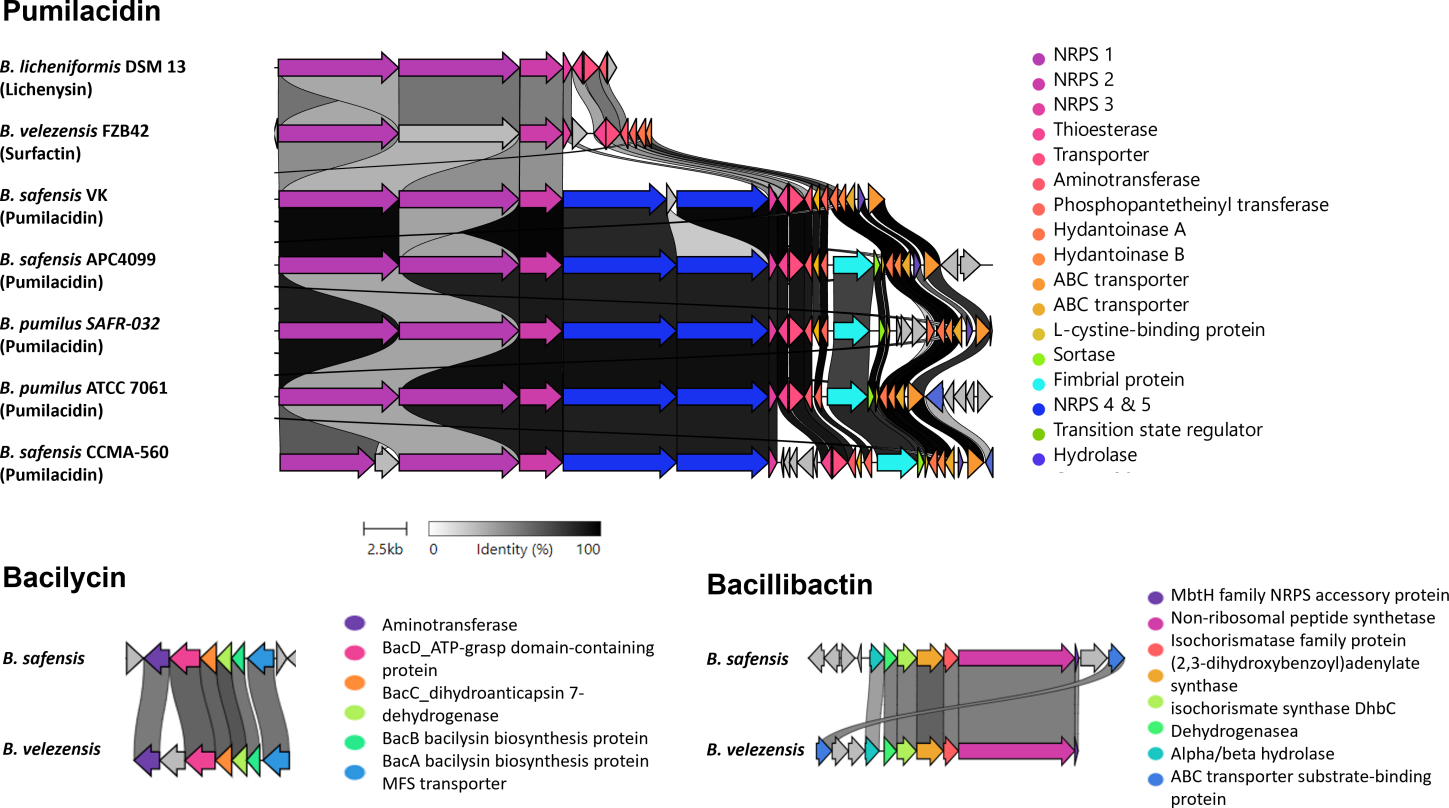
Schematic representation of the similarity between the gene clusters associated with lichenysin, bacilysin, and bacillibactin, and the identified gene clusters detected in *B. safensis* APC 4099 genome. Genes are color-coded according to the putative role of each protein.

Apart from the ribosomally-synthesized antimicrobial peptides, gene clusters coding for non-ribosomally-synthesized antimicrobial compounds were predicted using antiSMASH in the genome of *B. safensis* APC 4099. Specifically, three gene clusters with 98%, 85%, and 80% similarity to pumilacidin (64), bacilysin (52–54), and bacillibactin (51), respectively, were identified (Fig. 4; Table S3). The gene cluster showing 98% similarity with the pumilacidin gene cluster from *B. safensis* CCMA-560 included all the structural components of pumilacidin. Specifically, it was comprised of five biosynthetic core non-ribosomal peptidase synthetases (NRPS). In agreement with *B. safensis* CCMA-560 gene srfAA, the NRPS 1 gene encodes a 3570 amino acids protein, and is composed of 10 domains, which according to antiSMASH include three condensation domains, three adenylation domains, three thiolation domains, and one epimerization domain. These enzymes direct the assembly of the amino acids Glu-Leu-D-Leu into the peptide structure. The NRPS 2 gene encodes a 3,564 amino acids protein, which is two amino acids shorter than the *B. safensis* CCMA-560 gene srfAB. This protein is also comprised of three condensation domains, three adenylation domains, three thiolation domains, and one epimerization domain, which incorporate the amino acids Ile-Asp-D-Leu to the peptide. The NRPS 3 gene encodes a protein which is composed of 1,278 amino acids, one amino acid more than the product of the gene srfAC of *B. safensis* CCMA-560. The protein is composed of one condensation domain, one adenylation domain, one thiolation domain, and a thioesterase domain. This enzymatic cluster is involved in the addition of the amino acid isoleucine to the peptide chain. In agreement with the *B. safensis* CCMA-560 gene ORFx, the NRPS 4 gene encodes a 3,377 amino acids protein, composed of a ketoreductase and two condensation, one adenylation, one thiolation and one epimerization domain, the catalytic activity of which leads to the incorporation of the amino acid D-Val. Finally, the NRPS 5 gene encodes a 2,731 amino acids protein, which has an additional amino acid compared to the *B. safensis* CCMA-560 ORFy gene product. The protein is composed of condensation, adenylation, thiolation, and thioesterase domains. The enzymatic activity of these enzymes results in the addition of valine to the peptide. The gene cluster coding for pumilacidin from *B. safensis* APC 4099 was found to have lower sequence similarity with the biosynthetic gene cluster coding for pumilacidin produced by *B. safensis* VK (97% similarity), *B. pumilus* SAFR-032 (90% similarity), and *B. pumilus* ATCC 7061 (90% similarity). Additionally, the gene cluster was more distantly related to the lichenysin gene cluster from *B. licheniformis* DSM 13 (65), and the surfactin gene cluster from *B. velezensis* FZB42 (56) coding (56).

Compared to the *bac* operon coding for bacilysin, which was previously identified in *B. velezensis* FZB42, there was 63.50%, 74.04%, 68.77%, and 69.40% similarity with *bacA*, *bacB*, *bacC*, and *bacD* genes, respectively. These are the core genes implicated in bacilysin synthesis, with the *bacA*, *bacB*, and *bacC* gene products being predicted to be associated with anticapsin production, and the *bacD* gene to encode a ligase, which catalyzes the linkage between the anticapsin and alanine residues (66). Moreover, compared to the bacillibactin-associated cluster from *Bacillus* sp. WMMC1349, the bacillibactin variant from *B. safensis* APC 4099 had 58–71% similarity to the enzymes coding for bacillibactin, which include isochorismate synthase DhbC, (2,3-dihydroxybenzoyl) adenylate synthase, isochorismate synthase DhbC, 2,3-dihydro-2,3-dihydroxybenzoate dehydrogenase and amino acid adenylation domain-containing protein. Furthermore, a gene cluster with 53% similarity to fengycin from *B. subtilis* (56, 57) was detected. In addition to these ribosomally and non-ribosomally synthesized antimicrobial peptides, three gene clusters coding for the secondary metabolites, terpene and T3PKS, were identified.

### Bacteriocin homology analysis and structure prediction

**Fig 5:**
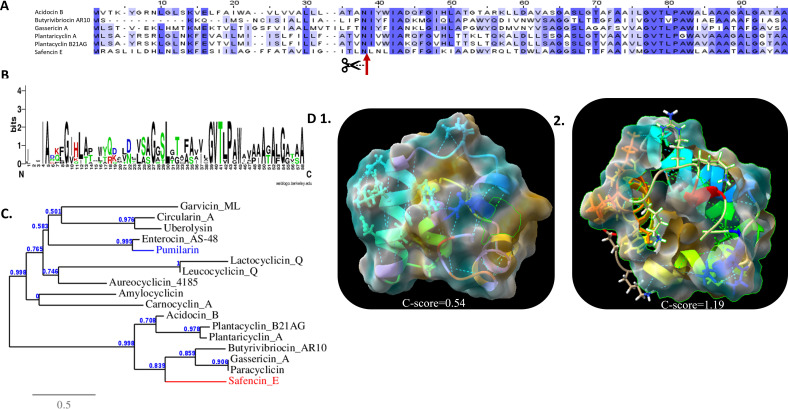
Structural characteristics of safencin E, and pumilarin. (**A**) Sequence alignment among the Class IIc circular bacteriocins acidocin B, butyrivibriocin AR10, gassericin A, plantaricyclin A, plantacyclin_B21AG, and safencin E, highlighting in blue the conserved amino acids. (**B**) Large letters in the Web Logo represent the conserved amino acids among the Class IIc circular bacteriocins acidocin B, butyrivibriocin AR10, gassericin A, plantaricyclin A, plantacyclin_B21AG, paracircularin, and safencin E. The hydrophobic, hydrophilic, acidic, and basic amino acids are shown in black, green, blue, and red, respectively. (**C**) Phylogenetic tree showing the similarity of safencin E with other identified circular bacteriocins based on the amino acid sequences of the mature peptide. Bar 0.5 indicates nucleotide substitutions per site. Safencin E and pumilarin are colored red and blue, respectively. (**D**) Predicted tertiary structures of safencin E and pumilarin. The degree of lipophilicity ranges from −20 to 0 to 20, and is colored blue-white-brown, respectively. Root mean square distance (RMSD) indicates the average distance of pairs of residues between the model and template. (D1) Safencin E comprises four α-helices structures, among which coil structures interfere (*C*-score = 0.54, TM-Score = 0.79 ± 0.09, RMSD = 1.9 ± 1.6 Å). (D2) Pumilarin is composed of five α-helices structures, among which coil structures interfere (*C*-score = 1.19, TM-score = 0.88 ± 0.07, RMSD = 1.1 ± 1.1 Å). Safencin E and pumilarin create a circular structure, according to which the hydrophobic amino acids hide inside the structure. The *C*-score and the TM-scores evaluate the global accuracy of the 3D structure model and the range from −5 to 2 and >−1.5, respectively, implies a model with correct global topology.

To evaluate the degree of homology of the identified bacteriocins within the *B. safensis* APC 4099 genome, namely plantazolicin, safencin E, and pumilarin, with other previously identified bacteriocins, an amino acid sequence comparison was carried out. Alignment among the identified 93-amino-acid safencin E precursor peptide with other circular bacteriocins, including gassericin A, butyrivibriocin AR10, acidocin B, plantaricyclin A, plantacyclin B21AG, and paracyclicin, identified the cleavage site of safencin E, separating the core 58-amino acid peptide from the leader peptide (Fig. 5A). Additionally, investigation into amino acid conservation within the amino acid sequences among the mature group ii circular bacteriocins revealed that the greatest degree of conservation occurred at the C-terminus of the peptides (Fig. 5B). The conserved amino acids in the C-terminus were mainly hydrophobic, including an amino acid motif composed of GVTL (or V)PA(or G)W. The N-terminal regions also contained several conserved hydrophobic amino acids. The presence of these residues suggests their possible involvement in the biogenesis and antimicrobial activity of this group of circular bacteriocins (67). Furthermore, evaluation of the amino acid sequence similarity of safencin E with other circular bacteriocins indicated a higher homology with the members of Group II compared to Group I circular bacteriocins (pumilarin was also included; Fig. 5C). Alignment between the plantazolicin gene identified in *B. safensis* APC 4099 with the plantazolicin gene described by Scholz et al. (45) indicated a six-amino acid difference in the leader peptide (Fig. S3). Similarly, the pumilarin gene indicated a one amino acid difference in the leader peptide with the pumilarin gene initially described for *B. pumilus* B4107 (46) (Fig. S4).

Regarding the structural conformation of the bacteriocins encoded by the strain *B. safensis* APC 4099, the predicted secondary structure of safencin E features four helical-bundle structures composed of α-helices, named α1 to α4, at residues 3–8, 14–25, 30–39, and 42–53, respectively (Fig. 5D). (i) The folding of the amphipathic helices is predicted to result in the development of a hydrophobic core, composed of the hydrophobic residues detected mainly in the α-helices α3 and α4. Similarly, pumilarin features helical-bundle structures consisting of five α-helices, named β1 to β5, at residues 3–5, 9–20, 25–35, 40–46, and 48–62, respectively. Additionally, a strand is created by the hydrophobic residues 67 and 68 (Fig. 5D). (ii) The hydrophobic core is predicted to be formed mainly by the hydrophobic amino acid residues 7–47. On the contrary, the mature 14 amino acid residue LAP (Linear azol(in)e-containing peptides) plantazolicin forms a linear secondary structure. The presence of the Cys and Ser/Thr residues is responsible for the post-translational modification that generates the thioether crosslinks known as (methyl)lanthionines (58).

### Colony mass spectrometry

**Fig 6:**
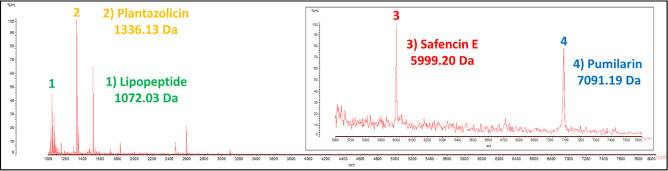
Colony MALDI-TOF mass spectrum showing the molecular masses of putative lipopeptide (1,072 Da), plantazolicin (1,336 Da), safencin E (5,999 Da), and pumilarin (7,091 Da).

MALDI TOF MS of peptides extracted from colonies detected putative lipopeptide (1,072 Da), plantazolicin (1,336 Da), safencin E (5,999 Da), and pumilarin (7,091 Da) masses (Fig. 6).

### HPLC analysis of antimicrobials produced by *B. safensis* APC 4099

As genome mining suggested that *B. safensis* APC 4099 has the ability to produce a number of antimicrobials, the presence of antimicrobial peptides in the CFS or their release from the cell pellet using IPA extraction was evaluated, with purification of the novel safencin E and pumilarin being of particular interest. The CFS and the IPA-treated cell pellet were initially partially purified using Strata C18-E SPE column extraction and the C18 SPE eluents were further fractionated by reversed phase HPLC. HPLC fractions were assessed for antimicrobial masses of interest by MALDI TOF MS and fractions of interest were tested for antimicrobial activity against *L. bulgaricus* LMG 6901 by WDA.

**Fig 7:**
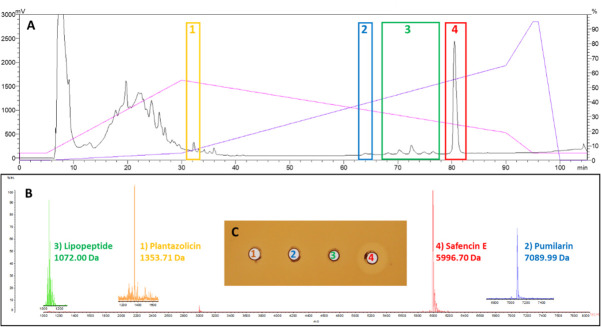
(A) HPLC chromatogram of the CFS of *B. safensis* APC 4099 showing elution of plantazolicin (1) at 31 min, pumilarin (2) at 64 min, lipopeptides (3) between 68 and 78 min and safencin E (4) at 81 min. (B) MALDI TOF MS of HPLC fractions showing the expected molecular mass for (1) plantazolicin (1,353 ± 3 Da modified 1,336 Da), (2) pumilarin (7,089.99 ± 3 Da, (3) lipopeptide 1,072 ± 3 Da, and (4) safencin E 5996.70 ± 3 Da. (C) Antimicrobial activity of HPLC fractions containing plantazolicin (1), pumilarin (2), lipopeptide (3), and safencin E (4) against *L. bulgaricus* LMG6901.

The HPLC chromatogram of the CFS of *B. safensis* APC 4099 shows elution of a number of peaks across the HPLC gradient with hydrophilic peptides eluting in the first 30 min and the more hydrophobic peptides eluting between 60 and 82 min (Fig. 7A). All fractions were assessed for antimicrobial masses of interest by MALDI TOF MS and masses correlating with the theoretical masses of the antimicrobials were detected for plantazolicin (1) at 31 min, pumilarin (2) at 64 min, lipopeptides (3) between 68 and 78 min, and safencin E (4) at 81 min (Fig. 7B). Specifically, a 1,353.71 Da mass was detected in the peak eluting at 31 min which correlates with the modified 1,354 Da (i.e., 1,336 ± 18 Da) mass reported for plantazolicin (45). The pumilarin mass (7,089.99 Da) was detected in the small peak eluting at 64 min (2) suggesting that pumilarin (7,087.34 ± 3 Da) is being produced in very small quantities. A series of masses with increasing 14 Da increments (1,058, 1,072, 1,086, and 1,100 Da), due to increasing methylation of the lipopeptide chain, were detected in peaks eluting between 68 and 78 min (Fig. S5). The possible addition of extra methylations increases the chain length and hydrophobicity of the molecules resulting in them eluting sequentially as the solvent content of the gradient increases. The 1,072 Da mass (3), putatively assigned to pumilacidin A, was detected in the highest peak eluting at 72 min. The masses 1,058, 1,086, and 1,100 Da were assigned to pumilacidin B, pumilacidin E, and pumilacidin C, respectively. Finally, a 5,996.70 Da mass (4) correlating with the safencin E theoretical mass (5,997.99 ± 3 Da) was detected in the dominant HPLC peak eluting at 81 min. Aliquots of the HPLC fractions containing plantazolicin (1), pumilarin (2), lipopeptide (3), and safencin E (4) were assayed against *L. bulgaricus* LMG 6901 and safencin E was shown to be active against this indicator (Fig. 7C). The safencin E-containing fraction also displayed activity against *L. lactis* HP (data not shown). The *B. safensis* APC 4099 cell extract was also assessed by reversed phase HPLC and interestingly, plantazolicin and lipopeptide yields were higher suggesting that these antimicrobials are cell-associated. Pumilarin was not present in the cell extract though safencin E was purified at levels similar to the CFS extract (data not shown).

## DISCUSSION

The deterioration of food quality due to spoilage and pathogenic microbes is a prime concern of the food industry. Considering that 1.3 billion tons of food appropriate for human consumption is lost or wasted yearly, developing solutions for a sustainable food future is critical (68, 69). According to the World Health Organization, 600 million people become infected, and 420,000 die from foodborne diseases annually (70). These numbers are expected to rise dramatically over the coming years, due to the rise of antibiotic resistance by several foodborne disease-causing strains (71). Additionally, the growing demand of consumers for naturally-preserved and minimally-processed foods raises the necessity for identification of biopreservatives, capable of preventing the growth of spoilage and pathogenic microbes in food products (72). Indeed, several LAB have been commercially applied as starter cultures for preserving food products and increasing product shelf-life (73–75).

The present study revealed the capability of the broad-spectrum antimicrobial-producing strain *B. safensis* APC 4099 isolated from bees' gut, to inhibit the growth of foodborne pathogens and spoilage microbes. Specifically, the strain’s CFS indicated strong anti-clostridial, anti-bacilli, anti-staphylococci, and anti-listeria activity, as well as effective anti-fungal activity against food contaminants *A. niger*, *P. variotii*, *Cladosporium* sp., *Geotrichum* sp., and *Byssochlamys nivea*. Whole-genome sequencing and genome mining analysis using BAGEL4 and antiSMASH revealed that the strain’s broad-spectrum antimicrobial activity was due to the production of several antimicrobials. Indeed, 7.16% of the genome is devoted to production of antimicrobial activity, which is higher than the estimated percentage for strains that belong to the *B. subtilis* group (4–5%) (76). These include a novel, Class IIc circular bacteriocin, named safencin E, ribosomally synthesised antimicrobial peptides plantazolicin and pumilarin, non-ribosomal peptides, including pumilacidin, bacilysin, and bacillibactin and secondary metabolites, terpene, and T3PKS.

Safencin E represents a novel member of the Class II circular bacteriocins raising the number of those characterized so far in Group II to 7 and the overall characterized circular bacteriocins to 25 (77–80). This group of circular bacteriocins is considered important for biotechnological applications. Their head-to-tail cyclization post-translational modification provides them with the advantage of having greater stability compared to linear peptides (81). Specifically, a covalent bond links the N- and C-termini, which makes these circular peptides resistant to various peptidases and proteases and provides them with temperature and pH stability (82). Another characteristic of these peptides is their high number of hydrophobic amino acid residues. Structural analysis of safencin E predicted that hydrophobic amino acid residues could be detected both inside, and on the surface of the structure. Also, multiple sequence alignment identified a conserved amino acid motif in the C-terminus of the group ii circular bacteriocins, composed of the amino acids GVT (L or V)P(A or G)W. The analysis revealed that the bacteriocin with the highest amino acid identity to safencin E is butyrivibriocin AR1044 (52.5%). Most circular bacteriocins are positively charged, unlike safencin E and butyrivibriocin AR1044, in that they are negatively charged. This observation suggests a mode of action different from that proposed for the other circular bacteriocins involving electrostatic interactions with the negatively charged bacterial membrane, which does not require a receptor molecule (13).

Generally, circular bacteriocins have been reported to exhibit broad-spectrum antimicrobial activity, including anti-clostridial and anti-listeria activity (13). The European Food Safety Authority and the European Centre for Disease Prevention and Control) reported listeriosis as the foodborne disease with the highest proportion of hospitalized cases for the Year 2020 (83). Therefore, identifying highly stable bioactive peptides against these pathogens is very important. A previous study indicated that pumilarin demonstrates activity against *S. aureus, B. cereus* ATCC 14579, and *E. faecalis* (46). Additionally, Xin et al. (21) revealed that another Class IIc circular bacteriocin, bacicyclicin XIN-1 was active against *B. cereus*, *L. monocytogenes*, and *S. aureus*. Moreover, *B safensis* APC 4099 was predicted to encode plantazolicin, a polyheterocyclic, linear ribosomally-synthesized and post-translationally modified peptide (45). Whole genome sequencing analysis allowed us to evaluate, based on the identified antimicrobial compounds, the spectrum of inhibition of the strain. Specifically, it was reported that plantazolicin has narrow-spectrum activity against other *Bacillus* species (59) and that pumilarin and the lipopeptides were active against *Listeria* and staphylococci (46, 84). Also, as reported by Perez et al. (13), that generally, circular bacteriocins, like the novel bacteriocin that was identified in the present study, named safencin E, have been reported to exhibit anti-clostridial and anti-listerial activity. Therefore, the lack of production of antimicrobial peptides that would inhibit the growth of gram-negative bacteria prevented us from testing other gram-negative bacteria, apart from *Escherichia coli* UCC, *Klebsiella pneumoniae* UCC, and *Pseudomonas aeruginosa* UCC, to demonstrate that the strain is not active against gram-negative bacteria.

Purification of the antimicrobials produced by the strain was challenging as the antimicrobials are optimally produced under different growth conditions, including temperatures and media composition. Growing the strain in TSB broth supplemented with 0.5% lactose under shaking conditions at 37°C was best suited for the production of safencin E and pumilarin, in particular. Lipopeptide, plantazolicin, pumilarin, and safencin E were purified from CFS, but higher levels of plantazolicin and lipopeptides were purified from the cell extract. The masses detected in the colony mass spectrum correlated well with the masses detected from the HPLC-purified antimicrobials with the exception of plantazolicin where an 18 Da increase was observed between the colony mass spectrum (1,336 Da) and the HPLC fraction (1,354 Da). This represents a modified version of plantazolicin (60) and appears during purification suggesting that the molecule is vulnerable to this modification. Plantazolicin was described previously in wild-type *B. amyloliquefaciens* FZB42 strain (60) and is produced by a 12-gene cluster with the precursor peptide undergoing post-translational modifications by the enzymes plantazolicin synthase, dehydrogenase, metalloprotease and methylotransferase. The 1,072 Da lipopeptide mass, is similar to pumilacidin A (1,072 Da), produced by *B. pumilus* strains (85). Pumilacidin A was described previously as a nonribosomally-synthesized biosurfactant created by a mixture of circular heptapeptides (Glu, Leu, D-Leu, Leu, Asp, D-Leu, and [(Leu/Ile)/Val]) which are connected to fatty acids of variable length (50, 84). A series of masses (1,058, 1,072, 1,086, and 1,100 Da) detected in *B. safensis* APC 4099 correlates with masses observed for pumilacidins A-E with increasing fatty acid chain length (84, 85). Purified safencin E has a mass of 5,996.70 Da supporting the assumption that safencin E has a 35-amino acid leader sequence and a 58-amino acid core peptide sequence, taking into consideration that a mass of 18 Da is lost due to head-to-tail circularization (6,016–18 = 5,998 Da).

Commonly, bacilli represent important biocontrol strains. Their spore-forming ability, combined with their capability to metabolize or assimilate various carbon sources, polymers, and several other substances, provides them with increased adaptive capability in diverse environmental conditions (86). The ability of *B. safensis* APC 4099 to produce multiple antimicrobials of different classes is highly desirable as it is likely to extend the inhibitory potential of the strain. *B. safensis* C3, a mung bean isolate, was also reported to contain three “bacteriocin biosynthetic systems” including plantazolicin, safencin E, and a single amino acid pumilarin variant they named safencin (Romero-Severson et al., 2021). Although the authors attempted to purify these compounds, the molecular mass of their purified antimicrobial (3.3 kDa) does not correlate with plantazolicin, safencin E, nor the pumilarin variant masses. Therefore, this study is the first report of purification of these antimicrobials from *B. safensis*. Owing to their enhanced anti-fungal activity, *B. safensis* has been applied as a biocontrol agent in previous bioprospecting studies (87–91). For instance, Sharma et al. (89) indicated that *B. safensis* strains isolated from the chickpea rhizosphere prevented *Sclerotinia sclerotiorum* growth by 62.41%. In agreement with our results, the researchers identified clusters coding for lipopeptides that have been described to possess anti-fungal activity, such as pumilacidin and fengycin (92). Biosurfactants, such as pumilacidin, have been shown to prevent biofilm formation in microbes, whereas fengycin distorts the cell membrane and causes pore formation, leading to cell death (93, 94). Additionally, pumilacidin is considered an important candidate for the remediation of heavy metals, improvement of oil recovery, and bioremediation performance in used engine oil (64, 95). Specifically, when applied in engine oil-contaminated sand, pumilacidin contributed to oil recovery by 39.78%. Also, this biosurfactant caused 82% and 100% removal of the heavy metals cadmium and lead, respectively. Our study revealed the presence of gene clusters related to the production of pumilacidin and fengycin. Regarding pumilacidin, *B. safensis* APC 4099 was found to produce a mixture of approximately four lipopeptides with molecular masses ranging from 1,058 to 1,110 Da (the highest peak was found at *m/z* 1,072 Da), which contain seven amino acids combined with a fatty acid chain of 13 and 18 carbons. Although the expression of pumilacidins A–E was shown, the expression of fengycin was not detected using MALDI-TOF MS analysis. However, the expression of lipoproteins is affected by several factors, including nutrients, as well as physiological and physiochemical features (96). Genome analysis also predicted the presence of bacilysin, an antimicrobial dipeptide that shows anti-bacterial and anti-fungal activity (97). Bacilysin is comprised of two amino acids, l-alanine and the uncommon amino acid l-anticapsin. It is believed to become activated after being inserted inside the microbial cells following hydrolysis by fungal peptidases, leading to the release of anticapsin (66). Anticapsin is a glutamine analog that inhibits the catalytic activity of aminotransferase of glucosamine-6-phosphate synthase (GFA). Catalytic activity of GFA is important for the initial step of hexosamine biosynthesis. Therefore, inhibition of its activity prevents the biosynthesis of fundamental structural macromolecules, such as peptidoglycan, glycoproteins, lipopolysaccharides, and chitin. The presence of bacilysin has been reported previously in other *B. safensis* strains (91). Overall, this study suggests that the observed antimicrobial activity of *B. safensis* APC 4099 is likely a result of the synergistic activity of lipopeptides, peptides, and other secondary metabolites.

### Conclusions

*B. safensis* APC 4099 is a strain with strong antimicrobial activity due to its ability to produce multiple antimicrobial compounds. The combination of genome mining, and MALDI-TOF MS analysis revealed the expression of two circular bacteriocins, a novel, Class IIc bacteriocin named safencin E, and pumilarin, the lantibiotic plantazolicin, and the lipopeptide pumilacidin, as possible candidates for the observed antimicrobial activity. However, the possibility that other antimicrobials and secondary metabolites also contribute cannot be excluded. These findings highlight the biotechnological importance of *B. safensis* APC 4099 bioactive compounds as biocontrol agents for food ecosystems against spoilage and pathogenic microbes and in agriculture against phytopathogens.

## Data Availability

The authors confirm all supporting data, code and protocols have been provided within the article or through supplementary data files.

## SUPPLEMENTAL MATERIAL

Supplemental material aem.01942-24-s0001.docx Figures S1 to S5; Tables S1 to S3.

ASM does not own the copyrights to Supplemental Material that may be linked to, or accessed through, an article. The authors have granted ASM a non-exclusive, world-wide license to publish the Supplemental Material files. Please contact the corresponding author directly for reuse.
