# Supplementary material for: Bacillus safensis APC 4099 has broad-spectrum antimicrobial activity against both bacteria and fungi and produces several antimicrobial peptides, including the novel circular bacteriocin safencin E
Source: Appl Environ Microbiol. 2024 Dec 31;91(1):e01942-24. doi: 10.1128/aem.01942-24 (PMC7617318; doi:10.1128/aem.01942-24)
Supplement: Supplemental material — Figures S1 to S5; Tables S1 to S3. [file aem.01942-24-s0001.docx]

**Supplementary Material**


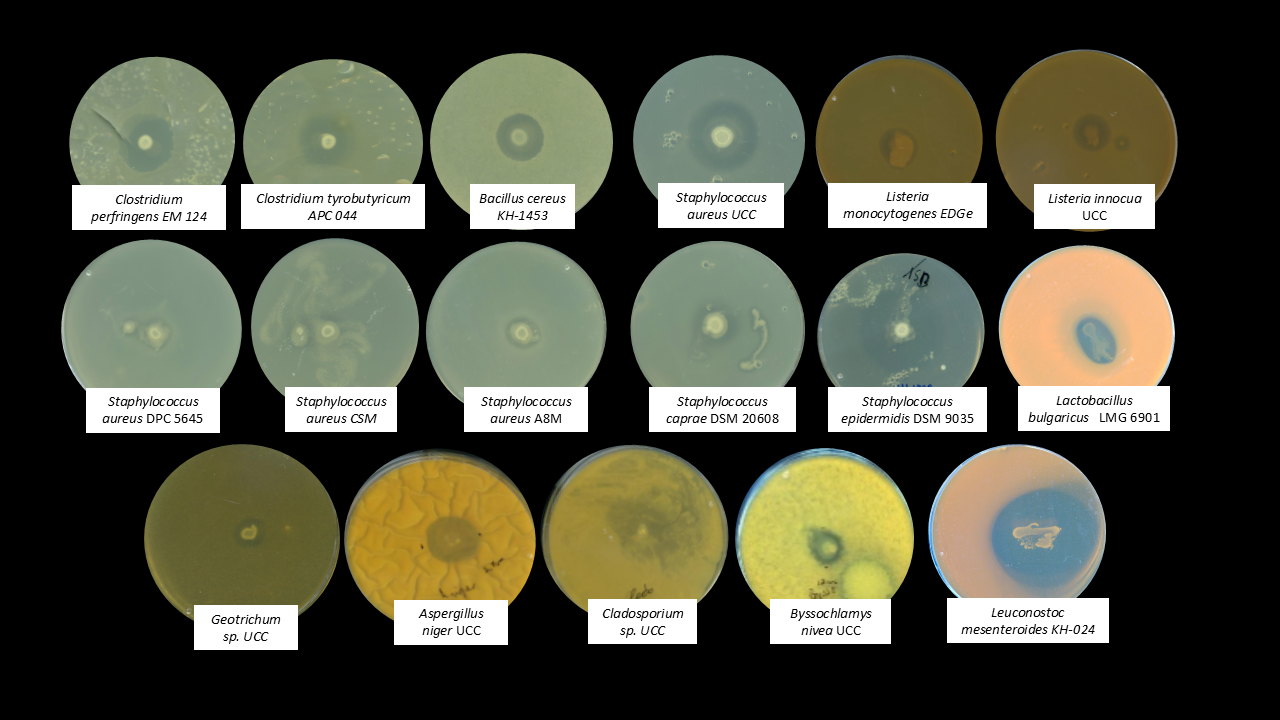


**Supplementary Figure S1.** Agar spot assay of *B. safensis* APC 4099 against a selection of bacterial and fungal indicators strains.


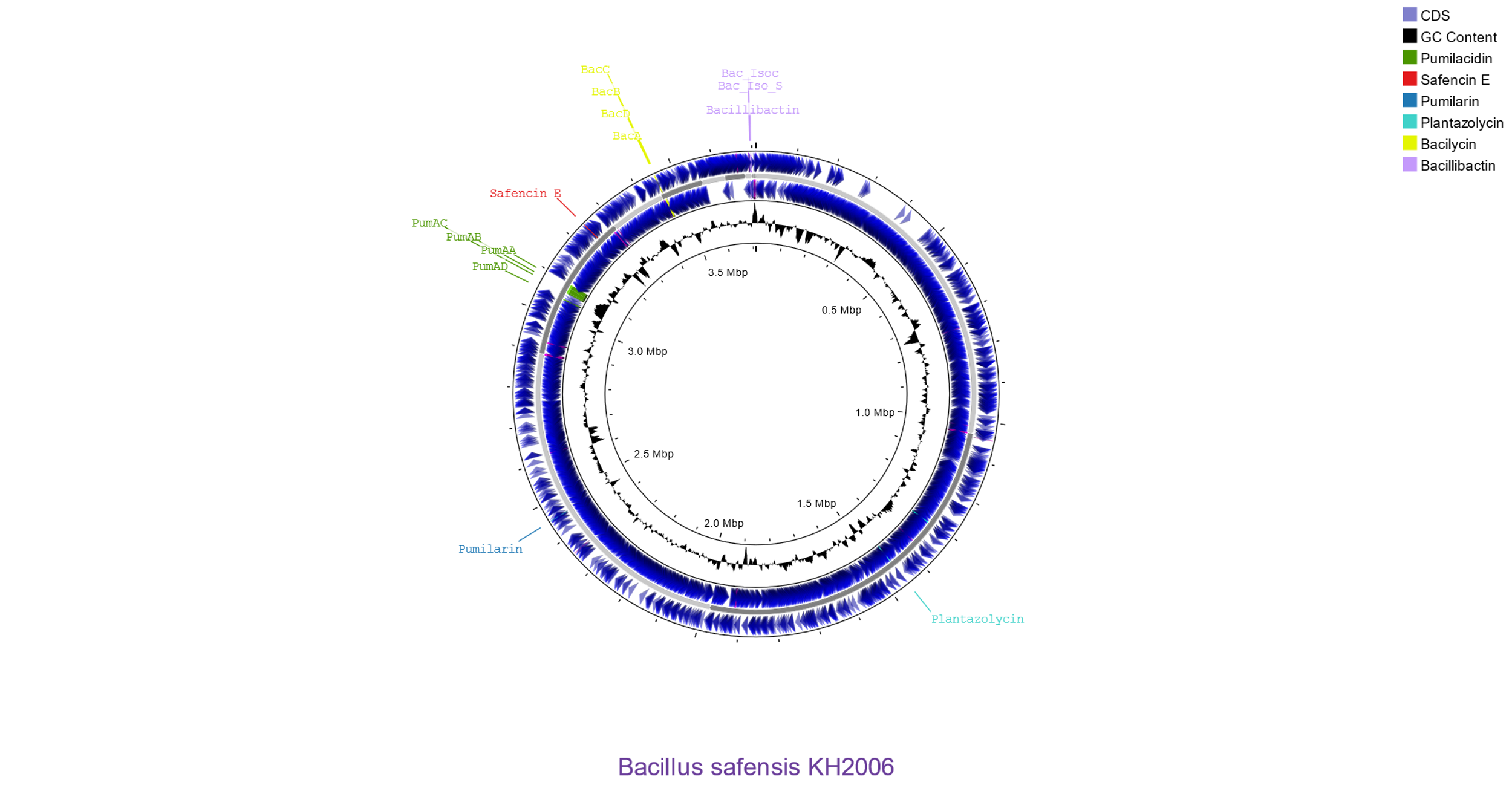


**Supplementary Figure S2.** Map of the gene sequences coding for the detected bacteriocins and non-ribosomally synthesized peptides across the circular genome of *B. safensis* APC 4099.


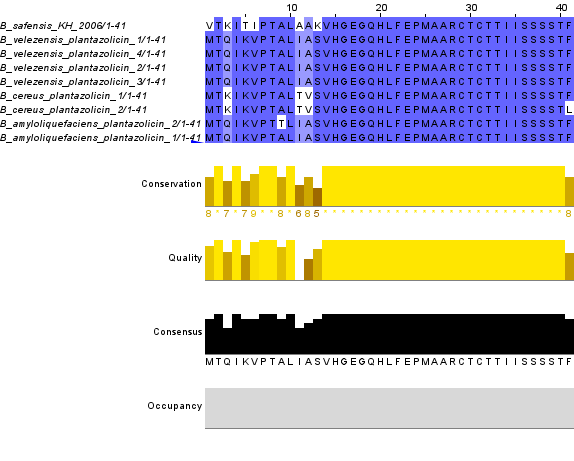


**Supplementary Figure S3.** Sequence alignment among plantazolicins identified from *B. safensis* (present study), and other Bacilli closest hinds identified following NCBI Blast, including *B. velezensis*, *B. cereus*, and *B. amyloliquefaciens,* including *B. amyloliquefaciens* FZB42 (41), highlighting with blue color the conserved amino acids.


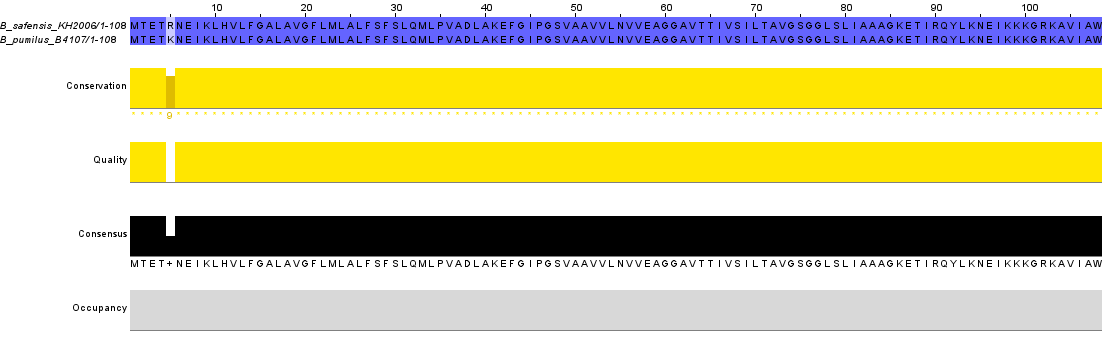


**Supplementary Figure S4.** Sequence alignment between pumilarin identified from *B. safensis* (present study), and *B. pumilus* B4107, highlighting with blue color the conserved amino acids.


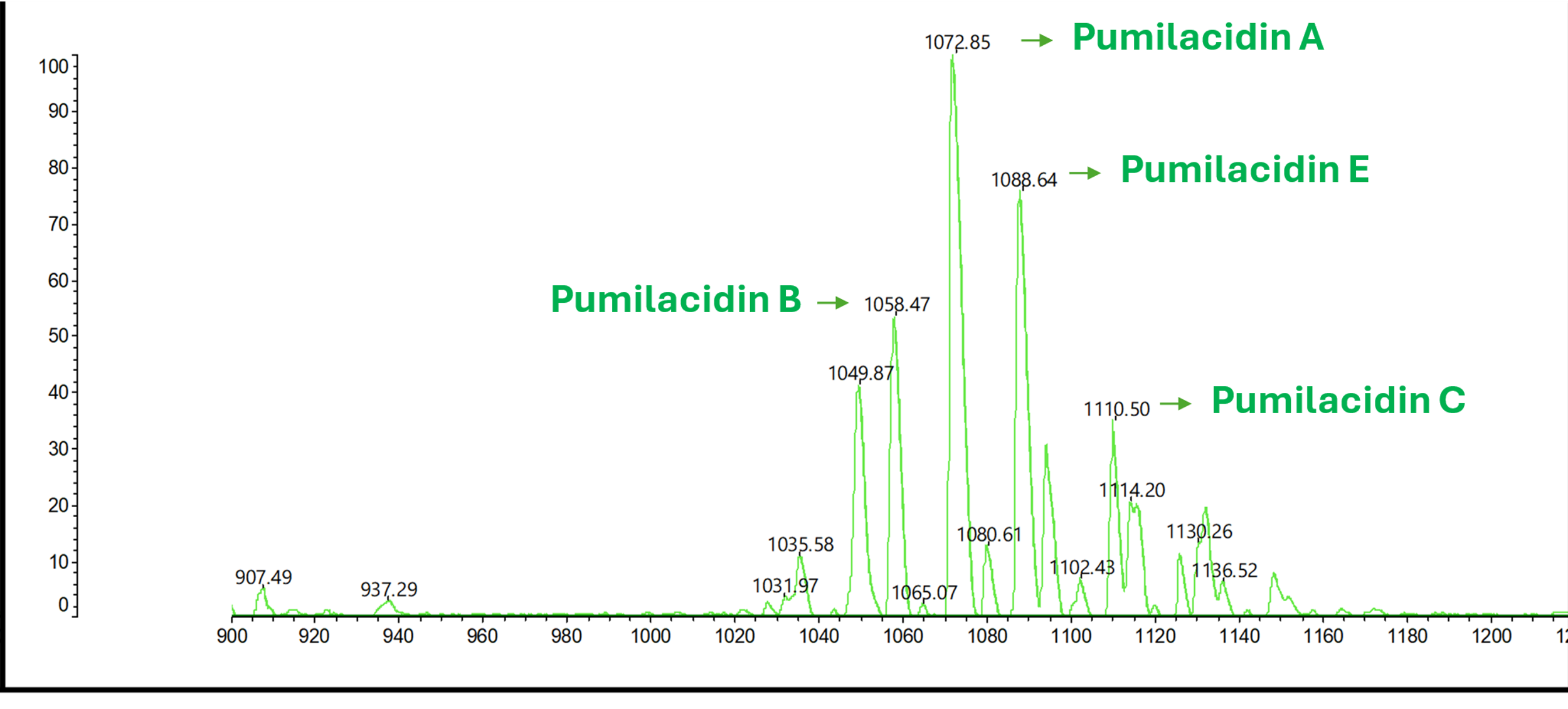


**Supplementary Figure S5.** MALDI TOF MS of HPLC fractions showing the molecular mass for lipopeptide isoforms ions.

**Supplementary Table 1**. Evaluation of the ability of the CFS to inhibit the growth of tested indicators in concentrations 5%, 10%, 25%, and 50% compared to control, based on LSD test.

| Microorganism | Time point | CFS concentration | Mean Difference (I-J) | Std. Error | Significant difference compared to control | 95% Confidence Interval | |
| --- | --- | --- | --- | --- | --- | --- | --- |
|  |  |  |  |  |  | Lower Bound | Upper Bound |
| *B. cereus* KH-1453 | 12 hours | 5% | 0.5236 | 0.01775 | <.001 | 0.484 | 0.5632 |
|  |  | 10% | 0.5248 | 0.01775 | <.001 | 0.4852 | 0.5644 |
|  |  | 25% | 0.52477 | 0.01775 | <.001 | 0.4852 | 0.5643 |
|  |  | 50% | 0.52467 | 0.01775 | <.001 | 0.4851 | 0.5642 |
|  | 24 hours | 5% | 0.6463 | 0.00942 | <.001 | 0.6253 | 0.6673 |
|  |  | 10% | 0.64743 | 0.00942 | <.001 | 0.6264 | 0.6684 |
|  |  | 25% | 0.64727 | 0.00942 | <.001 | 0.6263 | 0.6683 |
|  |  | 50% | 0.6471 | 0.00942 | <.001 | 0.6261 | 0.6681 |
|  | 48 hours | 5% | 0.5924 | 0.02544 | <.001 | 0.5357 | 0.6491 |
|  |  | 10% | 0.63647 | 0.02544 | <.001 | 0.5798 | 0.6932 |
|  |  | 25% | 0.63717 | 0.02544 | <.001 | 0.5805 | 0.6939 |
|  |  | 50% | 0.6373 | 0.02544 | <.001 | 0.5806 | 0.694 |
| *L. innocua* UCC | 12 hours | 5% | -0.12667 | 0.04232 | 0.014 | -0.221 | -0.0324 |
|  |  | 10% | 0.58333 | 0.04232 | <.001 | 0.489 | 0.6776 |
|  |  | 25% | 0.64333 | 0.04232 | <.001 | 0.549 | 0.7376 |
|  |  | 50% | 0.66333 | 0.04232 | <.001 | 0.569 | 0.7576 |
|  | 24 hours | 5% | -0.04667 | 0.08817 | 0.608 | -0.2431 | 0.1498 |
|  |  | 10% | 0.33 | 0.08817 | 0.004 | 0.1336 | 0.5264 |
|  |  | 25% | 0.56 | 0.08817 | <.001 | 0.3636 | 0.7564 |
|  |  | 50% | 0.61333 | 0.08817 | <.001 | 0.4169 | 0.8098 |
|  | 48 hours | 5% | -0.06 | 0.02556 | 0.041 | -0.117 | -0.003 |
|  |  | 10% | 0.17667 | 0.02556 | <.001 | 0.1197 | 0.2336 |
|  |  | 25% | 0.55333 | 0.02556 | <.001 | 0.4964 | 0.6103 |
|  |  | 50% | 0.57333 | 0.02556 | <.001 | 0.5164 | 0.6303 |
| *L. monocytogenes* EDGe | 12 hours | 5% | 0.36667 | 0.05203 | <.001 | 0.2507 | 0.4826 |
|  |  | 10% | 0.42667 | 0.05203 | <.001 | 0.3107 | 0.5426 |
|  |  | 25% | 0.453 | 0.05203 | <.001 | 0.3371 | 0.5689 |
|  |  | 50% | 0.45333 | 0.05203 | <.001 | 0.3374 | 0.5693 |
|  | 24 hours | 5% | 0.34333 | 0.06096 | <.001 | 0.2075 | 0.4792 |
|  |  | 10% | 0.16667 | 0.06096 | 0.021 | 0.0308 | 0.3025 |
|  |  | 25% | 0.47633 | 0.06096 | <.001 | 0.3405 | 0.6122 |
|  |  | 50% | 0.47667 | 0.06096 | <.001 | 0.3408 | 0.6125 |
|  | 48 hours | 5% | 0.02 | 0.05645 | 0.73 | -0.1058 | 0.1458 |
|  |  | 10% | 0.20333 | 0.05645 | 0.005 | 0.0776 | 0.3291 |
|  |  | 25% | 0.40667 | 0.05645 | <.001 | 0.2809 | 0.5324 |
|  |  | 50% | 0.41333 | 0.05645 | <.001 | 0.2876 | 0.5391 |
| *L.mesenteroides* KH-024 | 12 hours | 5% | 0.67 | 0.09437 | <.001 | 0.4597 | 0.8803 |
|  |  | 10% | 0.74333 | 0.09437 | <.001 | 0.5331 | 0.9536 |
|  |  | 25% | 0.72313 | 0.09437 | <.001 | 0.5129 | 0.9334 |
|  |  | 50% | 0.78397 | 0.09437 | <.001 | 0.5737 | 0.9942 |
|  | 24 hours | 5% | 1.14667 | 0.05522 | <.001 | 1.0236 | 1.2697 |
|  |  | 10% | 1.26 | 0.05522 | <.001 | 1.137 | 1.383 |
|  |  | 25% | 1.27 | 0.05522 | <.001 | 1.147 | 1.393 |
|  |  | 50% | 1.26667 | 0.05522 | <.001 | 1.1436 | 1.3897 |
|  | 48 hours | 5% | 0.56667 | 0.10433 | <.001 | 0.3342 | 0.7991 |
|  |  | 10% | 1.74 | 0.10433 | <.001 | 1.5075 | 1.9725 |
|  |  | 25% | 1.74333 | 0.10433 | <.001 | 1.5109 | 1.9758 |
|  |  | 50% | 1.74333 | 0.10433 | <.001 | 1.5109 | 1.9758 |
| *S. epidermitis* KM 1928 | 12 hours | 5% | 0.02583 | 0.00895 | 0.016 | 0.0059 | 0.0458 |
|  |  | 10% | 0.0263 | 0.00895 | 0.015 | 0.0064 | 0.0462 |
|  |  | 25% | 0.02583 | 0.00895 | 0.016 | 0.0059 | 0.0458 |
|  |  | 50% | 0.01707 | 0.00895 | 0.086 | -0.0029 | 0.037 |
|  | 24 hours | 5% | 0.17453 | 0.03503 | <.001 | 0.0965 | 0.2526 |
|  |  | 10% | 0.18023 | 0.03503 | <.001 | 0.1022 | 0.2583 |
|  |  | 25% | 0.18053 | 0.03503 | <.001 | 0.1025 | 0.2586 |
|  |  | 50% | 0.1738 | 0.03503 | <.001 | 0.0957 | 0.2519 |
|  | 48 hours | 5% | 0.30997 | 0.0332 | <.001 | 0.236 | 0.3839 |
|  |  | 10% | 0.38983 | 0.0332 | <.001 | 0.3159 | 0.4638 |
|  |  | 25% | 0.39733 | 0.0332 | <.001 | 0.3234 | 0.4713 |
|  |  | 50% | 0.39143 | 0.0332 | <.001 | 0.3175 | 0.4654 |
| *S. aureus* CSM | 12 hours | 5% | 0.09887 | 0.0247 | 0.003 | 0.0438 | 0.1539 |
|  |  | 10% | 0.11087 | 0.0247 | 0.001 | 0.0558 | 0.1659 |
|  |  | 25% | 0.11437 | 0.0247 | <.001 | 0.0593 | 0.1694 |
|  |  | 50% | 0.1356 | 0.0247 | <.001 | 0.0806 | 0.1906 |
|  | 24 hours | 5% | 0.11617 | 0.04056 | 0.017 | 0.0258 | 0.2065 |
|  |  | 10% | 0.17723 | 0.04056 | 0.001 | 0.0869 | 0.2676 |
|  |  | 25% | 0.22957 | 0.04056 | <.001 | 0.1392 | 0.3199 |
|  |  | 50% | 0.2559 | 0.04056 | <.001 | 0.1655 | 0.3463 |
|  | 48 hours | 5% | 0.15963 | 0.03631 | 0.001 | 0.0787 | 0.2405 |
|  |  | 10% | 0.32483 | 0.03631 | <.001 | 0.2439 | 0.4057 |
|  |  | 25% | 0.44877 | 0.03631 | <.001 | 0.3679 | 0.5297 |
|  |  | 50% | 0.4793 | 0.03631 | <.001 | 0.3984 | 0.5602 |
| *S. aureus* A8M | 12 hours | 5% | 0.1343 | 0.02351 | <.001 | 0.0819 | 0.1867 |
|  |  | 10% | 0.1472 | 0.02351 | <.001 | 0.0948 | 0.1996 |
|  |  | 25% | 0.14793 | 0.02351 | <.001 | 0.0956 | 0.2003 |
|  |  | 50% | 0.14737 | 0.02351 | <.001 | 0.095 | 0.1997 |
|  | 24 hours | 5% | 0.18787 | 0.04687 | 0.002 | 0.0834 | 0.2923 |
|  |  | 10% | 0.2608 | 0.04687 | <.001 | 0.1564 | 0.3652 |
|  |  | 25% | 0.2824 | 0.04687 | <.001 | 0.178 | 0.3868 |
|  |  | 50% | 0.2828 | 0.04687 | <.001 | 0.1784 | 0.3872 |
|  | 48 hours | 5% | 0.11423 | 0.05187 | 0.052 | -0.0014 | 0.2298 |
|  |  | 10% | 0.35513 | 0.05187 | <.001 | 0.2395 | 0.4707 |
|  |  | 25% | 0.46487 | 0.05187 | <.001 | 0.3493 | 0.5805 |
|  |  | 50% | 0.46607 | 0.05187 | <.001 | 0.3505 | 0.5817 |
| *S. aureus* UCC | 12 hours | 5% | 0.0008 | 0.10041 | 0.994 | -0.2229 | 0.2245 |
|  |  | 10% | 0.20303 | 0.10041 | 0.071 | -0.0207 | 0.4268 |
|  |  | 25% | 0.2022 | 0.10041 | 0.072 | -0.0215 | 0.4259 |
|  |  | 50% | 0.20263 | 0.10041 | 0.071 | -0.0211 | 0.4264 |
|  | 24 hours | 5% | -0.0532 | 0.0371 | 0.182 | -0.1359 | 0.0295 |
|  |  | 10% | 0.33777 | 0.0371 | <.001 | 0.2551 | 0.4204 |
|  |  | 25% | 0.3394 | 0.0371 | <.001 | 0.2567 | 0.4221 |
|  |  | 50% | 0.33977 | 0.0371 | <.001 | 0.2571 | 0.4224 |
|  | 48 hours | 5% | -0.02993 | 0.06945 | 0.676 | -0.1847 | 0.1248 |
|  |  | 10% | 0.30053 | 0.06945 | 0.001 | 0.1458 | 0.4553 |
|  |  | 25% | 0.4563 | 0.06945 | <.001 | 0.3015 | 0.6111 |
|  |  | 50% | 0.423 | 0.06945 | <.001 | 0.2682 | 0.5778 |
| *Cladiosporium* sp UCC | 24 hours | 5% | 0.03717 | 0.01295 | 0.017 | 0.0083 | 0.066 |
|  |  | 10% | 0.11437 | 0.01295 | <.001 | 0.0855 | 0.1432 |
|  |  | 25% | 0.1115 | 0.01295 | <.001 | 0.0826 | 0.1404 |
|  |  | 50% | 0.11553 | 0.01295 | <.001 | 0.0867 | 0.1444 |
|  | 48 hours | 5% | 0.0173 | 0.02026 | 0.413 | -0.0278 | 0.0624 |
|  |  | 10% | 0.066 | 0.02026 | 0.009 | 0.0209 | 0.1111 |
|  |  | 25% | 0.0765 | 0.02026 | 0.004 | 0.0314 | 0.1216 |
|  |  | 50% | 0.0609 | 0.02026 | 0.013 | 0.0158 | 0.106 |
| *Geotrichum* sp UCC | 24 hours | 5% | -0.0942 | 0.06235 | 0.162 | -0.2331 | 0.0447 |
|  |  | 10% | 0.04853 | 0.06235 | 0.454 | -0.0904 | 0.1875 |
|  |  | 25% | 0.20413 | 0.06235 | 0.008 | 0.0652 | 0.3431 |
|  |  | 50% | 0.27353 | 0.06235 | 0.001 | 0.1346 | 0.4125 |
|  | 48 hours | 5% | 0.24143 | 0.2163 | 0.29 | -0.2405 | 0.7234 |
|  |  | 10% | 0.59143 | 0.2163 | 0.021 | 0.1095 | 1.0734 |
|  |  | 25% | 0.81047 | 0.2163 | 0.004 | 0.3285 | 1.2924 |
|  |  | 50% | 0.97143 | 0.2163 | 0.001 | 0.4895 | 1.4534 |
| *P. variottii* UCC | 36 hours | 5% | 0.18793 | 0.02431 | <.001 | 0.1338 | 0.2421 |
|  |  | 10% | 0.32827 | 0.02431 | <.001 | 0.2741 | 0.3824 |
|  |  | 25% | 0.36343 | 0.02431 | <.001 | 0.3093 | 0.4176 |
|  |  | 50% | 0.36493 | 0.02431 | <.001 | 0.3108 | 0.4191 |
|  | 48 hours | 5% | 0.20453 | 0.05391 | 0.004 | 0.0844 | 0.3246 |
|  |  | 10% | 0.34963 | 0.05391 | <.001 | 0.2295 | 0.4697 |
|  |  | 25% | 0.4903 | 0.05391 | <.001 | 0.3702 | 0.6104 |
|  |  | 50% | 0.48873 | 0.05391 | <.001 | 0.3686 | 0.6088 |
| *A. niger* UCC | 24 hours | 5% | -0.0381 | 0.10789 | 0.731 | -0.2785 | 0.2023 |
|  |  | 10% | -0.7572 | 0.10789 | <.001 | -0.9976 | -0.5168 |
|  |  | 25% | 0.2282 | 0.10789 | 0.061 | -0.0122 | 0.4686 |
|  |  | 50% | 0.2513 | 0.10789 | 0.042 | 0.0109 | 0.4917 |
|  | 48 hours | 5% | -0.12557 | 0.12971 | 0.356 | -0.4146 | 0.1634 |
|  |  | 10% | -0.8761 | 0.12971 | <.001 | -1.1651 | -0.5871 |
|  |  | 25% | 0.1618 | 0.12971 | 0.241 | -0.1272 | 0.4508 |
|  |  | 50% | 0.30577 | 0.12971 | 0.04 | 0.0168 | 0.5948 |
| *P. variottii* UCC | 36 hours | 5% | -0.06137 | 0.08885 | 0.505 | -0.2593 | 0.1366 |
|  |  | 10% | -0.1042 | 0.08885 | 0.268 | -0.3022 | 0.0938 |
|  |  | 25% | 0.24103 | 0.08885 | 0.022 | 0.0431 | 0.439 |
|  |  | 50% | 0.24463 | 0.08885 | 0.02 | 0.0467 | 0.4426 |
|  | 48 hours | 5% | -0.15337 | 0.07738 | 0.076 | -0.3258 | 0.019 |
|  |  | 10% | -0.3548 | 0.07738 | 0.001 | -0.5272 | -0.1824 |
|  |  | 25% | 0.3301 | 0.07738 | 0.002 | 0.1577 | 0.5025 |
|  |  | 50% | 0.3463 | 0.07738 | 0.001 | 0.1739 | 0.5187 |
| *B. nivea UCC* | 36 hours | 5% | -0.06137 | 0.08885 | 0.505 | -0.2593 | 0.1366 |
|  |  | 10% | -0.1042 | 0.08885 | 0.268 | -0.3022 | 0.0938 |
|  |  | 25% | 0.24103 | 0.08885 | 0.022 | 0.0431 | 0.439 |
|  |  | 50% | 0.24463 | 0.08885 | 0.02 | 0.0467 | 0.4426 |
|  | 48 hours | 5% | -0.15337 | 0.07738 | 0.076 | -0.3258 | 0.019 |
|  |  | 10% | -0.3548 | 0.07738 | 0.001 | -0.5272 | -0.1824 |
|  |  | 25% | 0.3301 | 0.07738 | 0.002 | 0.1577 | 0.5025 |
|  |  | 50% | 0.3463 | 0.07738 | 0.001 | 0.1739 | 0.5187 |

**Supplementary Table 2**. Comparison of the whole genome of *B. safensis* APC 4099 against other 10 *B. safensis* complete circular genomes

| **Column1** | ***B. safensis*_AHB11** | ***B. safensis*_H31R-08** | ***B. safensis*_BRM1** | ***B. safensis*_LG01** | ***B. safensis*_F6** | ***B. safensis*_BS-10L** | ***B. safensis*_APC_4099** | ***B. safensis*_PgKB20** | ***B. safensis*_ZK-1** | ***B. safensis*_SRCM125915** | ***B. safensis*_KCTC** |
| --- | --- | --- | --- | --- | --- | --- | --- | --- | --- | --- | --- |
| *B. safensis*_AHB11 | 1 | 0.97609 | 0.9868 | 0.985 | 0.9657 | 0.976 | 0.987503 | 0.976734 | 0.9767 | 0.96586 | 0.9760827 |
| *B. safensis*_H31R-08 | 0.9761 | 1 | 0.9749 | 0.976 | 0.967 | 0.986 | 0.974627 | 0.987193 | 0.98722 | 0.96726 | 0.9856386 |
| *B. safensis*_BRM1 | 0.9868 | 0.97494 | 1 | 0.988 | 0.9659 | 0.975 | 0.990647 | 0.975382 | 0.97553 | 0.96616 | 0.9751614 |
| *B. safensis*_LG01 | 0.9854 | 0.97617 | 0.9882 | 1 | 0.9659 | 0.976 | 0.988162 | 0.976865 | 0.97692 | 0.96602 | 0.976164 |
| *B. safensis*_F6 | 0.9657 | 0.96696 | 0.9659 | 0.966 | 1 | 0.967 | 0.965418 | 0.966207 | 0.96674 | 0.98984 | 0.9666697 |
| *B. safensis*_BS-10L | 0.9764 | 0.98571 | 0.9753 | 0.976 | 0.9666 | 1 | 0.974912 | 0.985934 | 0.9873 | 0.96679 | 0.9881202 |
| *B. safensis*_ APC_4099 | 0.9875 | 0.97463 | 0.9906 | 0.988 | 0.9654 | 0.975 | 1 | 0.975361 | 0.97528 | 0.96558 | 0.9740364 |
| *B. safensis*_PgKB20 | 0.9767 | 0.98719 | 0.9754 | 0.977 | 0.9662 | 0.986 | 0.975361 | 1 | 0.98841 | 0.96642 | 0.9858055 |
| *B. safensis*_ZK-1 | 0.9767 | 0.98722 | 0.9755 | 0.977 | 0.9667 | 0.987 | 0.975282 | 0.988413 | 1 | 0.96686 | 0.9870227 |
| *B. safensis*_SRCM125915 | 0.9659 | 0.96726 | 0.9662 | 0.966 | 0.9898 | 0.967 | 0.965582 | 0.966419 | 0.96686 | 1 | 0.9667215 |
| *B. safensis*_KCTC | 0.9761 | 0.98564 | 0.9752 | 0.976 | 0.9667 | 0.988 | 0.974036 | 0.985805 | 0.98702 | 0.96672 | 1 |

**Supplementary Table 3**. Similarity among genes of the gene cluster coding for *Bacillus safensis* APC 4099 lipopeptide compared to the gene cluster coding for pumilacidin, produced by *B. safensis* CCMA-560, *B. safensis* VK, *B. pumilus* SAFR-032, and *B. pumilus* ATCC 7061.

| Gene product | *B. safensis* CCMA-560 (pumilacidin) | *B. safensis* VK  (pumilacidin) | *B. pumilus SAFR-032*  (pumilacidin) | *B. pumilus* ATCC 7061 (pumilacidin) |
| --- | --- | --- | --- | --- |
| NRP synthetase | 98% | 97% | 90% | 90% |
| NRP synthetase 2 | 98% | 97.5% | 90% | 90% |
| NRP synthetase 3 | 98% | 98% | 91% | 92% |
| NRP synthetase 4 | 97% | 96% | 90% | 90% |
| NRP synthetase 5 | 97% | 97% | 90% | 90% |
